# A system dynamics model of the impact of COVID-19 pandemic and foreign direct investment in the global supply chain

**DOI:** 10.1186/s43093-022-00155-3

**Published:** 2022-09-24

**Authors:** Mojtaba Hajian Heidary

**Affiliations:** Department of Industrial Management, Faculty of Management and Accountancy, Allameh Tabataba’i University, West End Hemmat Highway, Dehkadehye-Olympic, Tehran, 1489684511 Iran

**Keywords:** Foreign direct investment (FDI), Global supply chain, System dynamics (SD)

## Abstract

COVID-19 pandemic is a great challenge that the world has been faced in recent decades. The pandemic has affected the global trade and has caused a dramatic fall in Foreign Direct Investments (FDI). The impact on FDI is concentrated in the countries that are severely hit by the pandemic, although negative impact of disruptions is observed in other countries. In this paper, a system dynamics model is developed to simulate the impact of the pandemic on the FDI. Results showed that the level of force majeure condition and also the level of flexibility are two important factors that impact on the FDI and other important variables of the supply chain. In the case of lower severity of pandemic and higher flexibility, FDI is higher than the case that these parameters are in their worst condition.

## Introduction

Foreign direct investment (FDI) is a type of investment that usually multinational companies made it in the countries. FDI can stimulate the economic growth and also improve the total income of the host country, especially when it goes to the developing countries. FDI is a key source of external finance for developing countries. On the other hand, a large portion of the world trade transactions occurs within global supply chain. FDI is a main driver of global supply chain. Hence, investigating the mechanism of the FDI in global supply is very important especially for developing countries. Policy makers face a dilemma whether FDI would be encouraged or prevented. FDI is suitable for the economic growth but also causes an unintended income inequality. Therefore, decisions regarding the FDI could not be made without a modeling procedure. Foreign direct investment (FDI) has become the necessity of every nation as it does accelerate growth in an economy [1, 2]. FDI is often vulnerable to economic situation and various types of disruptions. Since 2004, FDI flows to developed countries arrived at the lowest level, while, it is quite stable with an increase of around two percent for developing economies [3].

Most of the decisions made in global supply chains could be categorized at the strategic level. Also because of the disruptions, uncertainties in the demand, production, and import/export practices, global supply chain operations are usually consider as a complex process. The pandemic has also been caused additional complexities in the global trade.

**Fig. 1:**
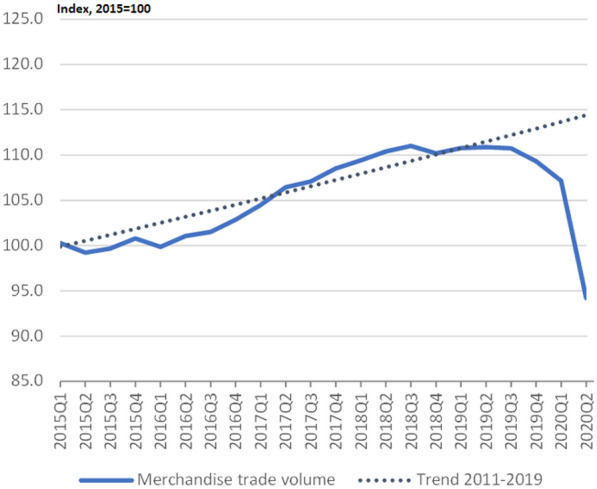
World merchandise trade volume. Source: WTO

The recent pandemic has caused serious economic consequences in the global supply chains. Based on the reports published by World Trade Organization (WTO) the global trade fell by 8.9 percent in 2020. In addition, the pandemic affected services trade more than merchandise trade. The global services trade fell by more than 20 percent in 2020. However, the impact of the pandemic shock on the global trade was different across countries. Figure 1 shows the negative impact of pandemic on the merchandise trade.

The severity of the effects of the pandemic in the host country have a negative impact by reducing investment capital and the number of investors. On the other hand, the damage caused by pandemic in the home country may encourage outward FDI [4]. In order to face with such disasters, increasing the flexibility of the supply chain would be beneficial [5, 6].

During the pandemic, developed and developing economies has been passing through a critical period, affecting all economic indicators of the countries and international trade. For developed economies, the loss of GDP was 7.8 percent and for developing economies it was around 2 percent. The loss of GDP globally as a consequence of pandemic was around 9 trillion dollars during 2020–2021 [7]. The effect of the pandemic on poor communities across the world has been derived 49 million individuals into extreme poverty and a reduced workforce across all economic sectors [8]. These facts highlights the importance of modelling different impacts of the pandemic.

Global financial crisis such as the pandemic perpetuates a sudden and sharp rise in the borrowing costs of developing countries [9]. Therefore the need for more FDI is seen in such economies. The implication of Covid-19 can also be seen on supply chains. The effect of disrupted supply chain has been seen mostly in the countries that are highly import dependent and it leads to inflationary pressure on the prices. These linkages between Covid-19, FDI and supply chain could be modeled to make the better decisions in such conditions.

In this paper we model the impact of the pandemic on the FDI and the global supply chain operations. One tool that can model this impact, is the system dynamics (SD). System dynamics method by focusing on the cause-and-effect relationships between the factors of the system, analyzes the problem and shows that how the interactions among the factors create a problem [10]. System dynamics is the best tool for understanding the behavior of complex systems. This approach focuses mainly on the idea that a system could be illustrated as the interactions, feedbacks, and delays among the components [11]. Hence, we use this approach to model the interactions of a global supply chain considering FDI and then simulate the model to achieve the results. Additionally, we consider the impact of the pandemic by a force majeure factor in the model. The main research question of this paper is: How system thinking approach can help decision makers to predict the outcomes of their actions in the global SCM?

The remainder of this paper is organized as follows: in the section "Literature review" we review the previous related literature to the problem. In the section "Method" preliminaries of the SD are presented. The simulation modeling framework is described in the section "Problem definition". In the next section, numerical results of the simulation are presented. Finally, some discussions about the results are presented in the section "Results of the simulation" and then some concluding remarks are presented in the last section.

## Literature review

Many researchers investigated the impact of the pandemic on different businesses in the last year. Some of them studied the future of the outbreak, such as [12]. Most of them used simulation approach to model and analyzed the mechanism of the outbreak. Simulation as a powerful tool in the stochastic conditions could be used to analyze and predict a system behavior over time [13]. Three main simulation techniques could be considered: Agent based simulation, discrete event simulation and System Dynamics. System dynamics is mainly used at the strategic analysis of the systems.

The system dynamics approach is an important tool to simulate the supply chain, even before the recent pandemic. Bhushi and Javalagi [14] categorized the applications of SD approach in the supply chain management and explained that international supply chain management is an important field of research that should be considered by the researchers. Sundarakani et al. [15] developed a global supply chain with four stages and analyzed the dynamics between its variables by using the SD approach. They investigated and simulated different scenarios of the economy. They concluded that information delays have important role in the global supply chain management.

Disruptions in the supply chain is another important problem of global supply chain management. Choi et al. [16] by using the system dynamics approach surveyed an automobile manufacturer and showed that the postponement strategy is a way to prevent the adverse effect of the disruptions. Nedelciu et al. [17] developed a SD model for the global phosphorus supply chain. Their model divided the world into eight regions. Social, economic and environmental dynamics play important roles in the model. The model was run up to 2050 to assess the need to phosphorus in each region.

During the recent pandemic, some of the researchers were attracted to investigate the impact of the outbreak on the supply chain operations using different simulation approaches. Some of them used SD approach. Sinha et al. [18] developed a system dynamics framework to simulate the impact of the COVID-19 pandemic on the supply chain. They compared pre- and post-pandemic situations. Their results showed that supply chain policies should not be same in these situations. Wang et al. [19] developed a system dynamics model of the hog market in china to predict the impact of pandemic on the supply chain. They showed that the impact of transportation disruptions is short-term. They also showed that delay has an important role in supply chain operations. Hajian Heidary [20] presented a model to analyze the impact of COVID-19 pandemic on the global supply chain operations. He showed that improving the flexibility of production capacity is an important strategies to cope with the pandemic negative impacts in the global supply chain.

The effect of foreign investment on the economic growth has been investigated remarkably. As a result, we can conclude that it contributes to the economic growth of the local country [21, 22]. This economic growth is due to an increase in employment, wages, and productivity of the local firms [23]. The effect of FDI on the country-level employment differs based on the development level of the host country. FDI in developing countries can create more jobs whereas this fact in the developed countries may be reversed [24, 25]. FDI also increases the per capita income of individuals in host countries. Studies have concluded that the foreign firms offer higher wages than the local firms [26–28]. FDI may also impacts on the economy level of the host country by improving the productivity of the local firms through technology transfer and technology spillover [29, 30].

Teekasap [31] by using the system dynamics approach, studied the process of foreign direct investment. In this study, two regions with separated economic situation were considered and FDI selection was based on the operating costs of the regions.

Global supply chains considering FDI, need a strategic arrangement between different components of the supply chain in different zones considering interactions between them, feedbacks and delays of the operations. Analysis of this system is complex and requires the use of system dynamics approach. In addition, the pandemic situation adds to the complexity of the issue.

To the best of our knowledge, there is no research in the literature that surveyed the impact of the pandemic and FDI on the global supply chain operations.

## Method

**Fig. 2:**
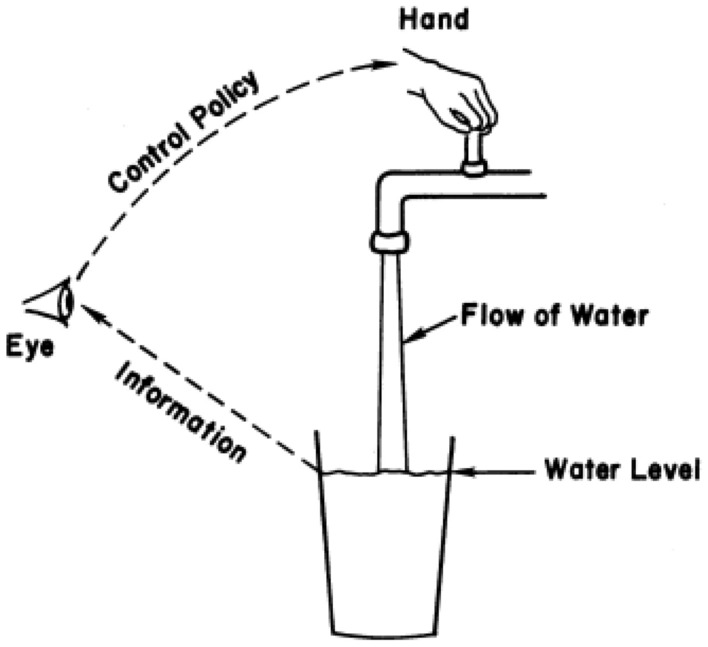
The process of decision making in SD [32]

Before continuing into the details of the problem, it is important to present the basic principles of system dynamics. System dynamics is a method that mainly used to analyze the complex systems. It is an approach to understand nonlinear relations of stock and flow variables of the system and also feedback loops that inherently exists in the system. Decision making process in SD involves three parts: (1) “consequences” that are needed to be achieved, (2) “actions” that should be done to get to the consequences and (3) “information loops” that connects these two parts. Forrester [32] illustrated this process as shown in the Fig. 2.

The process of decision making in the SD can be illustrated as filling a glass of water. It needs a control mechanism on the amount of water that flowing into the glass. It is a feedback loop from the level of water in the glass to eye to hand to control the level of water in the glass. In this simple example, the consequence that is to be achieved could be assumed as a certain amount of water in the glass. The action that is needed to get the consequence is to open the water tap.

Indeed, in a SD approach, the above mentioned process is applied to analyze the mechanisms of the system and to get results of different strategies. Afterwards, in order to identify the internal feedbacks of the system, a mental model (known as causal loop diagram) is depicted and then the stock and flow diagram is obtained.

## Problem definition

**Fig. 3:**
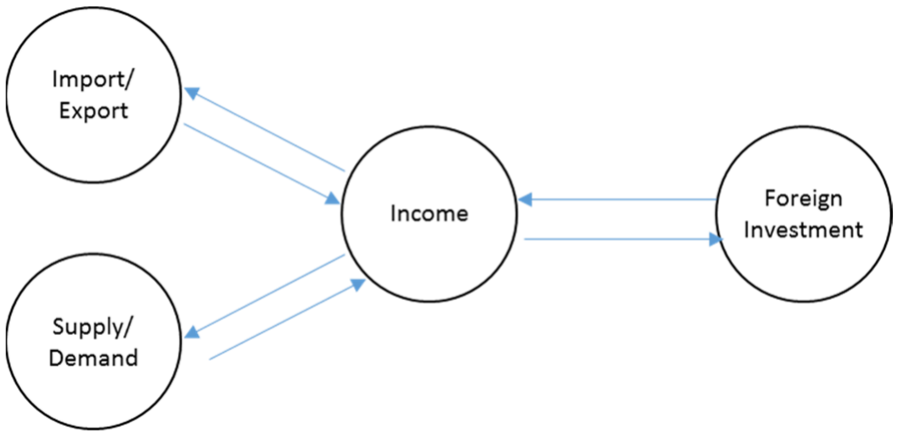
Relationships between the basic subsystems

In this paper the impact of COVID-19 pandemic and foreign direct investment in the global supply chain is surveyed. Our model consists of four subsystems: Local Supply/Demand, Import/Export, income and Investment. Figure 3 depicts the general relations between these subsystems:

Figure 3 implies that the import and export practices impact on the income. Changes of the income affect the ability of expansion of the production and the ability of response to the demand. In addition, foreign investment increases the income and FDI can stimulate economic growth which in turn affects the income.

Assumptions considered in this paper are as follows:

- The simulation length is 100 months.
- A normal good is considered in the supply chain.
- Lead time is known and constant.
- Import and export are distributed uniformly in each period.
- Global factors include exchange rate, tariff, import and export practices.
- Price elasticity, income elasticity, exchange rate, tariff are considered as constant.
- The capacity of production is finite and by increasing the income capacity of production is expanded.
- The pandemic condition is considered as the force majeure factor that impacts income, supply, import and price. This factor is treated as a trend instead of a constant value.
- All of the foreign investments are defined as a variable named FDI.

In this paper, based on the Hajian Heidary [20], underlying interrelations among the important variables of the global supply chain are discussed.

### Relationships between demand/supply and import/export variables

**Fig. 4:**
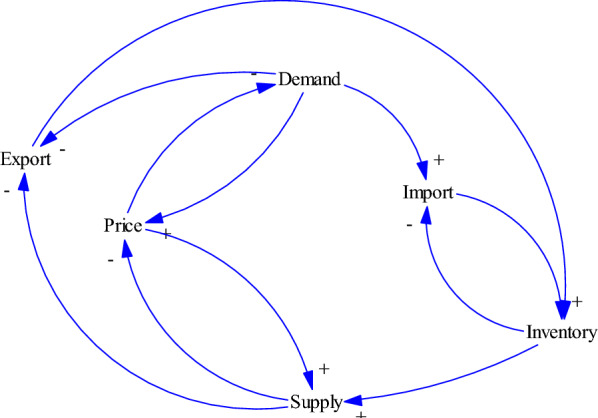
Relationships between demand/supply and import/export variables

Figure 4 depicts the basic relations between demand/ supply and import/ export variables in the model. The law of demand states that buyer’s demand will decrease when the price tends to rise and vice versa. The law of supply also states that at higher prices, sellers will tend to supply more of the products. These two laws specify the price of a product in a market. Changes in demand impact the rate of import or export in the economy and also the level of inventory to response to the market demand. A higher level of inventory enables a supply chain to supply more to the market.

### Relationships of the income variable with other subsystems of the model

**Fig. 5:**
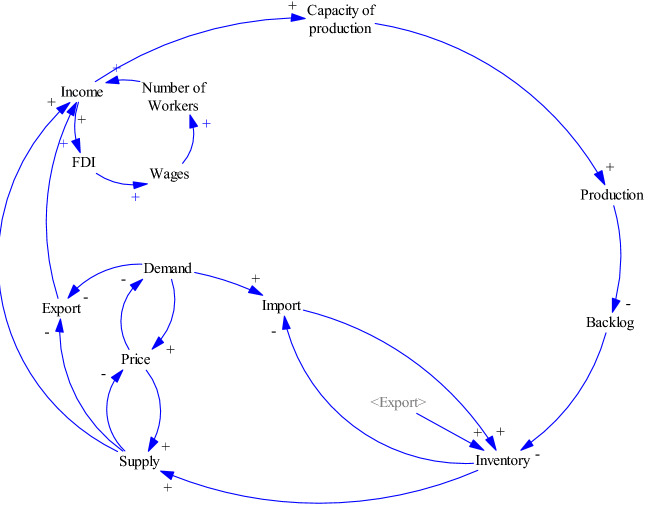
Relationships of the income variable with other subsystems of the model

The income earned from the export and selling of the products to the market is used to expand the production capacity and in turn, increases the rate of production. It is worthwhile to note that the expansion of production capacity may take some periods of time (delay time). The amount of workload that exceeds the capacity of production determines the backlog. Thus, the expansion of production decreases backlogs and consequently increases the inventory. On the other hand, when the foreign firms invest in the region, they usually offer a higher level of wage and therefore they will provide more jobs for people in that region. The higher number of workers creates more incomes for the region. These relationships are demonstrated in the Fig. 5.

### Relationships of order related variables with other subsystems

**Fig. 6:**
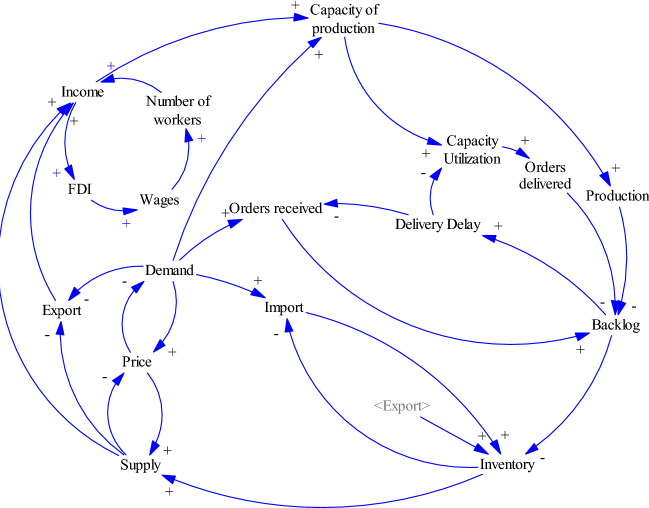
Relationships of order related variables with other subsystems

A higher level of backlog increases the delivery delay and in turn, results in dissatisfaction of the customers. The amount of orders received from the dissatisfied customer will decrease during the time. A higher level of backlog also decreases the capacity utilization. A lower amount of capacity utilization decreases the supply chain ability to on time delivery of orders. The complete model is illustrated in the Fig. 6.

COVID-19 pandemic has an adverse impact on the supply chain operations during the last two years.

Koshimura and Shuto [33] surveyed the Tsunami as a force majeure disaster condition in the supply chain. In our model, we define a force majeure parameter in order to determine the impact of the pandemic on the income, price, demand, rate of production and supply.

The force majeure factor is defined as a time based trend. The value of this parameter is assumed between 0 and 1 (worst and best conditions). This factor depicts the impact of force majeure conditions on the state of the business. Based on Sinha et al. [18], we assumed that a business fall during the beginning periods of simulation, then gradually the business condition is recovered and then due to the return of the disease the business condition will drop down to a lower level and then the recovery of business condition will continue until the end of the simulation.

**Fig. 7:**
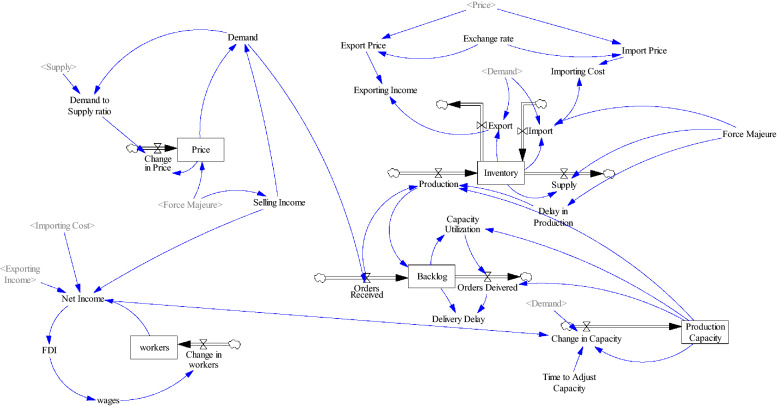
stock-flow diagram

The stock—flow diagram of the proposed model is illustrated in the Fig. 7. In this diagram there are five stock variables that together show the state of the system in each period. Also tracking some variables such as FDI and import/ export could be important in the analysis of the model.

In the next section, results of the simulation runs are presented.

## Results of the simulation

The SD model presented in the previous section is a basic framework of our simulations. The model should be examined for the accuracy and the robustness. In this paper, a scenario-based testing method is used to compare the results of the model in different situations with the expected outcome in a real situation. Parameters of the model are defined based on the reports published by the Iranian Ministry of Industry, Mine and Trade. In this section, results of simulating the proposed model in two scenarios are presented. An important parameter of the model is the force majeure factor. We consider two scenarios (Table 1) for the value of this parameter: scenario 1 (a random value between 0.5 and 0.9) or scenario 2 (a random value between 0.1 and 0.5).

**Table 1:** Scenarios of the force majeure factor

	Force majeure factor
Scenario 1	U(0.5, 0.9)
Scenario 2	U(0.1, 0.5)

Note that a low value of this factor is not desirable and indicates the worst business condition. Also, some sensitivity analyses are provided to get more insights into the problem.

**Fig. 8:**
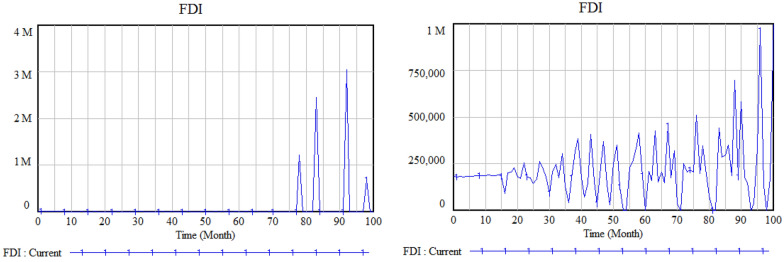
Changes in FDI in the worst case (left) and best case (right) of force majeure factor

In this section we compare two scenarios of the force majeure factor in the model. Results of the value of FDI in the worst and best case of force majeure factor are depicted in the Fig. 8.

As shown in the Fig. 8, in the worst case of the impact of pandemic on the businesses FDI is usually zero. But in the best case, FDI gradually improves as the simulation proceeds. It could be concluded that the impact of the pandemic on the businesses has a meaningful effect on the value of FDI.

Table 2 shows the simulation results (important variables of the model) of the scenario 1 and scenario 2.

**Table 2:** Simulation results of scenario analysis based on the 95% confidence interval

	FDI			Net income			Import			Export		
	Max (M)	Avg. (M)	Min	Max (M)	Avg. (M)	Min (M)	Max (M)	Avg. (M)	Min (M)	Max (M)	Avg. (M)	Min (M)
Scenario 1	7	4	0	100	74	15	3	1.7	1	4	3.2	1
Scenario 2	1	0.5	0	16	9	0	2	1.5	0	1.5	0.9	0

Results of the Table 1 shows that in the case of high value for the force majeure factor (scenario 1), FDI is higher, much more income can be earned, and in average, more import and export would be occurred rather than the scenario 2.

One of the parameters of the model is “time to adjust the capacity” that shows the flexibility of the supply chain to response to the disruptive events. The basic value of this parameter is considered as 1 month. In order to analyze the impact of the different values of this parameter on the model we defined two other values for this parameter: 0.5 month and 2 months that means high flexibility and low flexibility respectively. Table 3 shows the results of simulation regarding these cases.

**Table 3:** Simulation results of different flexibility of supply chain based on the 95% confidence interval

Scenario	Flexibility	FDI			Net income			Import			Export		
		Max (M)	Avg. (M)	Min	Max (M)	Avg. (M)	Min (M)	Max (M)	Avg. (M)	Min (M)	Max (M)	Avg. (M)	Min (M)
1	Low	5.7	3	0	125	85	10	2.5	1.5	0.7	2	1.7	0.5
	Basic	7	4	0	100	74	15	3	1.7	1	4	3.2	1
	High	7.5	4.2	0	91	66	25	3.1	1.8	1.2	4	3.5	1
2	Low	0.8	0.4	0	15	8	0	2	1.3	0	1	0.5	0
	Basic	1	0.5	0	16	9	0	2	1.5	0	1.5	0.9	0
	High	1.2	0.6	0	19	12	0	2	1.4	0	1.5	1	0

It could be concluded from the results of Table 3 that more flexibility would result higher investments, more income and also in average higher values for import and export.

Therefore, the level of force majeure condition and also the level of flexibility are two important factors that impact on the FDI and other important variables of the supply chain.

**Fig. 9:**
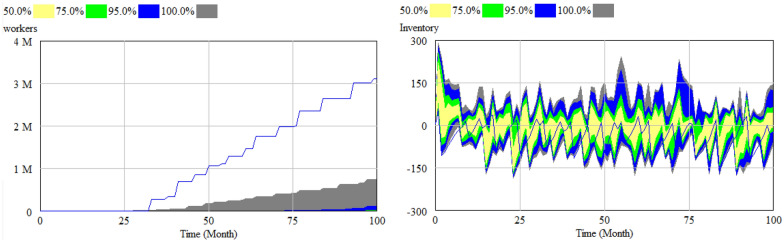
Sensitivity analysis of the number of “workers” and “inventory”

Number of workers and inventory are two stock variables that are important in this model. The results of the sensitivity analysis based on the different values of the force majeure factor (U(0,1)) and Time to adjust capacity (U(0.5,2)) are depicted in the Fig. 9.

Figure 9 shows that the number of workers in the worst case for pandemic and lowest level of flexibility will not exceed 1 million and also inventory will not exceed 300 units. These insight could help the decision makers to make the better decisions regarding their supply chains.

## Discussions

The Covid-19 pandemic has caused unprecedented changes in the worldwide trade. One of the main consequences of the pandemic was the disruptions in the supply chains. Designing more flexible supply chains and more flexible production capacity could help the firms to resist against these disruptions. On the other hand, the FDI increases the per capita income in the host countries. Also, more FDI flows into the host countries drive the wages up. Based on the results obtained in the previous section, in the case of lower severity of pandemic and higher flexibility, FDI is higher than the case that these parameters are in their worst condition. It highlights the importance of FDI in order to face with disruption in the global supply chains.

The results of this study could be beneficial to practitioners who are related to the foreign investment. Practitioners can use the results of this paper to better understanding the process of the global supply chain in disaster conditions like the pandemic. The results are also beneficial to scholars in the field of foreign investment and economic development. In addition, the model developed in this study could be used to test the different policies in the global supply chain during the pandemic or any same disasters.

## Conclusions

The recent pandemic made remarkable challenges for global trade. Availability of the products and services have been decreased during the last two years, and therefore, most of the firms have faced difficulties to response to demands. On the other hand, the complexity of global supply chain operations will enhance the process of decision making. Delivery delays are the other adverse consequences of the pandemic. Hence, system dynamics is the best approach to simulate this complex system. In this paper, the impact of the pandemic on the operations of global supply chain and FDI was modelled by using system dynamics approach. Also, the impact of the recent pandemic on the supply chain operations was modelled by a force majeure factor. This factor was defined as a time based trend that depicts the impact of the pandemic on the state of the business. Also, a scenario analysis was performed to indicate the simultaneous impact of the force majeure factor and delivery delay. Results showed that the level of force majeure condition and also the level of flexibility are two important factors that impact on the FDI and other important variables of the supply chain. In the case of lower severity of pandemic and higher flexibility, FDI is higher than the case that these parameters are in their worst condition. As a suggestion for the future researchers, analyzing the risk mitigation strategies in the supply chain operations could be considered. In addition, investigating the income inequality and the risk of internal conflict that are related to the FDI are the other suggestions for the future studies.

## Data Availability

Not applicable.

## Abbreviations

FDI: Foreign direct investment
SCM: Supply chain Management
SD: System dynamics
